# A Machine Learning System to Indicate Diagnosis of Idiopathic Pulmonary Fibrosis Non-Invasively in Challenging Cases

**DOI:** 10.3390/diagnostics14080830

**Published:** 2024-04-17

**Authors:** Yousef Ahmad, Joshua Mooney, Isabel E. Allen, Julia Seaman, Angad Kalra, Michael Muelly, Joshua Reicher

**Affiliations:** 1 Department of Pulmonary and Critical Care, University of Cincinnati Medical Center, 231 Albert Sabin Way, ML 0564, Cincinnati, OH 45267-0564, USA; 2 Stanford Health Care, Department of Pulmonary, Allergy, and Critical Care Medicine, 300 Pasteur Drive, Stanford, CA 94305, USA; 3 Department of Epidemiology & Biostatistics, University of California San Francisco, 550 16th Street, 2nd Floor, San Francisco, CA 94158-2549, USA; 4 Bay View Analytics, 6924 Thornhill Dr, Oakland, CA 94611, USA; julia@bayviewanalytics.com; 5 IMVARIA, 2930 Domingo Ave #1496, Berkeley, CA 94705, USA

**Keywords:** interstitial lung disease, pulmonary fibrosis, artificial intelligence, machine intelligence

## Abstract

Radiologic usual interstitial pneumonia (UIP) patterns and concordant clinical characteristics define a diagnosis of idiopathic pulmonary fibrosis (IPF). However, limited expert access and high inter-clinician variability challenge early and pre-invasive diagnostic sensitivity and differentiation of IPF from other interstitial lung diseases (ILDs). We investigated a machine learning-driven software system, Fibresolve, to indicate IPF diagnosis in a heterogeneous group of 300 patients with interstitial lung disease work-up in a retrospective analysis of previously and prospectively collected registry data from two US clinical sites. Fibresolve analyzed cases at the initial pre-invasive assessment. An Expert Clinical Panel (ECP) and three panels of clinicians with varying experience analyzed the cases for comparison. Ground Truth was defined by separate multi-disciplinary discussion (MDD) with the benefit of surgical pathology results and follow-up. Fibresolve met both pre-specified co-primary endpoints of sensitivity superior to ECP and significantly greater specificity (*p* = 0.0007) than the non-inferior boundary of 80.0%. In the key subgroup of cases with thin-slice CT and atypical UIP patterns (*n* = 124), Fibresolve’s diagnostic yield was 53.1% [CI: 41.3–64.9] (versus 0% pre-invasive clinician diagnostic yield in this group), and its specificity was 85.9% [CI: 76.7–92.6%]. Overall, Fibresolve was found to increase the sensitivity and diagnostic yield for IPF among cases of patients undergoing ILD work-up. These results demonstrate that in combination with standard clinical assessment, Fibresolve may serve as an adjunct in the diagnosis of IPF in a pre-invasive setting.

## 1. Introduction

ILD represents a spectrum of architectural and inflammatory lung diseases, with more than 200 different subtypes [1]. IPF is the most common of the ILD subtypes and, historically, has been associated with poor clinical prognosis, which has since been improved with antifibrotic therapies [2]. IPF patients are typically diagnosed in their 60s, with incidence and prevalence varying from 1 to 13 and 3 to 45 per 100,000, respectively [3]. Given its idiopathic nature, no discrete cause is recognized for the development of the disease, though a variety of factors, including male sex, age, cigarette smoking, family history, certain genetic mutations, and more, carry an elevated risk for the development of the condition. Many patients exhibit lengthy periods of asymptomatic or minimally symptomatic development of the disease but rates of progression from early signs of interstitial lung abnormality (ILA), especially as assessed by CT scans, into ILD or IPF are generally variable and low, leaving questions remaining regarding the best steps for the screening and monitoring of potential early signs of disease. IPF itself is characterized by rapidly progressive loss of lung function in a restrictive pattern but the vague early symptoms make delays in diagnosis common. In addition, the differentiation of IPF from other forms of ILD can be a challenge. Once IPF is diagnosed, the mainstays of treatment are oral antifibrotic therapies, shown to delay the progression of loss of lung function, and supportive care, including oxygen supplementation when necessary. Registries have demonstrated gastrointestinal side effects, including nausea, vomiting, and diarrhea, as the most common and important tolerability concerns with antifibrotic therapies [4]. Otherwise, numerous ongoing clinical trials continue to search for new therapies for IPF. Lung transplantation is generally reserved only for certain subpopulations of amenable patients.

In patients undergoing work-up for ILD, a combination of UIP patterns by computed tomography (CT) and concordant clinical findings define the non-invasive diagnosis of IPF [5]. Otherwise, tissue sampling, including surgical biopsy, may be indicated, though such tests carry substantial morbidity and mortality risks [6]. Although diagnostic guidelines exist, the subjective nature of clinical assessments and radiographic imaging interpretation leads to limitations in establishing an accurate diagnosis. Diagnostic agreement between clinicians is reported only as fair, with an estimated κ = 0.331 [7]. While subspecialized pulmonologists with ILD expertise offer moderately improved diagnostic agreement relative to clinicians with less experience, delayed access to tertiary referrals/centers of excellence in ILD remains and has been reported as a median of ~2.2 years, broadly limiting the overall value of such expertise [8]. Tertiary care access is further delayed in publicly insured and at-risk groups [9]. With respect to the contribution of CT imaging assessment to diagnosis, the UIP pattern specifically by CT has an estimated sensitivity of 14.3% (CI: 6.2–24.6%) and a specificity of 90.2% (CI: 77.9–99.0%) for predicting pathologic UIP by surgical biopsy, as calculated from the core referenced study in the ATS Guidelines [10,11]. In addition, inter-reader variability is high in the application of the imaging guidelines in assessing chest CTs, with a κ of only 0.36–0.42, even among ILD experts, despite the intended standardization [12]. The ATS 2022 Guidelines Update was formally expanded to allow “probable” UIP patterns as sufficient for diagnosis in selected subpopulations, though even here, issues remain, including concerns of spectrum bias in data collection and studies demonstrating applications in practice resulting in very wide variations in sensitivity and specificity [13,14]. Pathology-based genomics classification has been reported to have a sensitivity of 70% and a specificity of 87% for pathologic UIP, with the challenge of requiring the performance of invasive transbronchial biopsy sampling, limiting uptake [15,16]. Consequently, thought leaders and patient advocacy groups have repeatedly highlighted the need for “improved diagnostic tools that increase the speed and accuracy of diagnosis and facilitate early therapeutic intervention” [17].

Machine learning algorithms have shown promise in a variety of clinical use cases in assessing lung diseases [18]. Multiple complex features outside of the current standard imaging criteria have been identified via hand-crafted algorithm research and quantitative assessment of images in ILD that contain additional clinical value, suggesting that automation may improve diagnostic efficiency [19]. Additionally, early work investigating such algorithms in assessing ILD cases has shown promise, suggesting that CT evaluation by deep learning algorithms can serve effectively as an adjunct in the classification of fibrotic lung disease [20]. More recently, research has correlated the application of deep learning analysis with pathologic results and long-term outcomes [21,22].

The development and validation process of a new machine learning-based software tool, Fibresolve (IMVARIA Inc., Berkeley, CA, USA), was previously described, including the system’s potential for improvements in the non-invasive diagnosis of IPF [23]. Here, we further investigate the performance of Fibresolve via a multiple clinical panels study, assessing cases that went on to surgical biopsy to drive a more definitive diagnosis. The purpose of this study is to demonstrate the potential clinical value of the tool’s predictions of final IPF diagnosis from analysis of initial CT scans, especially in those cases without a typical UIP pattern. Specifically, this study is designed to assess the sensitivity and specificity of Fibresolve in pre-invasive diagnosis of IPF in an all-comers ILD population, with subsequent Ground Truth supported by surgical biopsy with histopathology, and to compare performance to clinicians in a pre-invasive setting. In addition, this study is designed to assess the diagnostic yield of Fibresolve in cases with atypical UIP patterns, providing an assessment of potential efficacy as a “digital biopsy” (i.e., non-invasive digital diagnostic) tool.

## 2. Materials and Methods

Our study compared the diagnostic performance of Fibresolve to multiple panels of clinicians in differentiating IPF from other types of ILD. This study followed “retrospective-prospective” study design (in line with National Cancer Institute definitions), re-analyzing prospectively collected cases with outcomes blinded to tool developers and clinical panels. Patient data cohorts originated from a large international dataset, including registries and previously completed clinical trials from 2005 to 2018. From the larger dataset, two US sites were selected. Data elements included demographics, medical history, medications, clinical questionnaires, clinical notes, and MDD records. Reference Standard Diagnosis (i.e., Ground Truth) was assigned by the local team and confirmed by the central site investigator, with the final diagnosis being adjudicated by MDD with surgical pathology results and clinical history. Assessments by new clinical panels as well as the Fibresolve model were then obtained using information collected at the baseline patient assessment (i.e., time zero), with comparison to the Ground Truth/Reference Standard Diagnosis to calculate performance measures.

**Figure 1:**
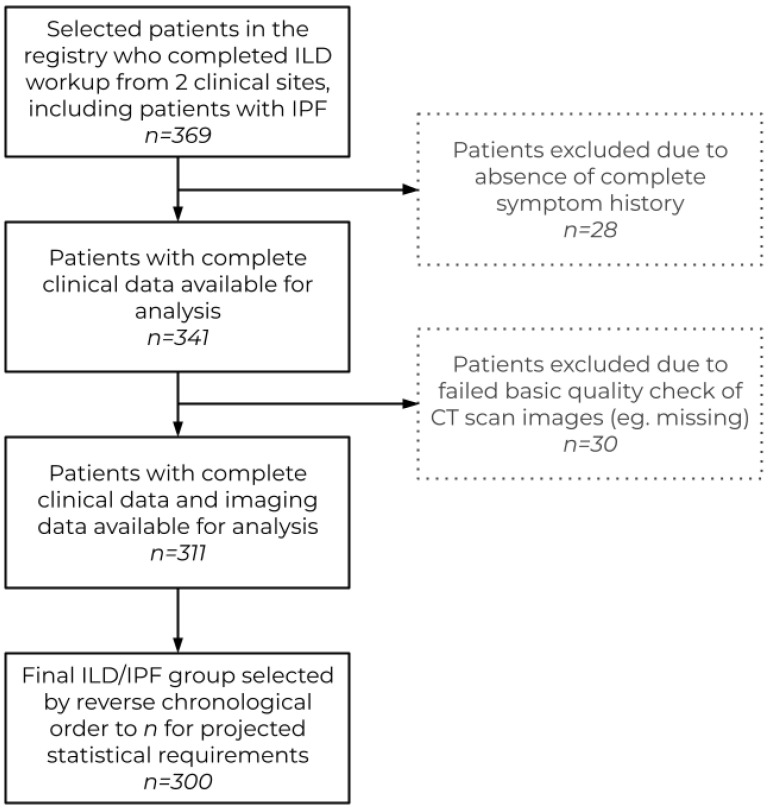
Inclusion and exclusion workflow used for case selection for completion of the panel study.

Inclusion criteria for the study included age > 18 years old, availability of complete symptom history, and CT scan with contiguous 1 to 5 mm axial slices with a full view of the lungs. A total of 369 cases were available from the two sites, of which 300 were selected based on the inclusion criteria and target sample size (Figure 1). The key target subgroup of interest was the group of cases with thin-slice (i.e., diagnostic) CT and atypical UIP patterns.

### 2.1. The Fibresolve Classifier: Development and Initial Validation

The Fibresolve classifier is a cloud-based software system including a data ingestion pipeline and machine learning classifier. The system is designed to analyze CT imaging in cases of suspected ILD for which IPF is a diagnostic consideration and predict final diagnosis as confirmed by final clinical assessment with surgical biopsy and/or follow-up. The system was developed with a >2000 patient dataset and is trained to recognize complex patterns correlated with a final diagnosis of IPF. The system assesses for a constellation of features beyond those that classically define UIP to facilitate the diagnosis of IPF in cases without a typical UIP pattern. During development, the algorithm is fed the entire CT volume and analyzes it in true 3D simultaneously. As previously reported, at the selected and optimized operating point, validation set sensitivity was 67% with a specificity of 90%, and the algorithm cutoff was set and locked during development.

### 2.2. Clinical Panel Reviews

An Expert Clinical Panel (ECP) of three subspecialized pulmonologists and two thoracic radiologists experienced in ILD reviewed all 300 cases together from timepoint zero, blinded to surgical pathology, long-term outcomes, and final diagnosis. The ECP was instructed to follow the then-active 2018 ATS Guidelines for positively confirming cases of IPF. Three additional smaller clinical panels (CPs), including one pulmonologist and one radiologist, each reviewed a representative 50-case subset sample to assess for inter-clinician variability with varying levels of experience. All cases were reviewed in an online case review system, with CT images displayed alongside all pre-invasive clinical, demographic, laboratory testing, pulmonary function testing, and symptom questionnaire information. The clinicians did not have access to the results from the machine learning classifier.

### 2.3. Fibresolve Classifier Results

**Figure 2:**
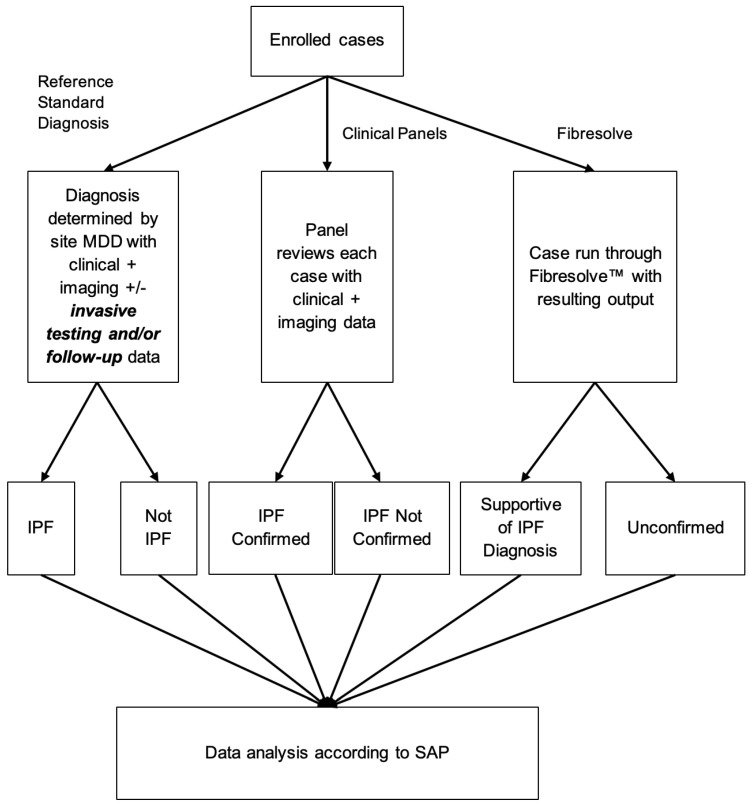
Methodological algorithm providing an overview of the architecture of the machine learning technology.

The Fibresolve classifier was run for each case at timepoint zero with results recorded in a locked database. The classifier analyzed only the CT images and did not analyze other clinical, demographic, or laboratory data (Figure 2). Of note, CT scan protocols varied significantly at timepoint zero, as not all patients were prospectively known to have ILD at the initial assessment point, and thus included thick-slice CT scans and protocols not necessarily optimized for assessment of the lungs.

### 2.4. Statistical Plan

This study’s endpoints were pre-specified in a complete Statistical Analysis Plan as part of a Clinical Study Protocol. Statistical analyses were performed in Stata^®^ software (17.1) and SPSS (Version 27). Comparisons between Fibresolve and the reference standard were examined using Binomial, Wald, and Score tests for proportions. The primary endpoints of the study were a specificity greater than a pre-selected 80% target threshold and a sensitivity of Fibresolve that was non-inferior or superior to the ECP, using one-sided 95% confidence intervals. The 80% specificity boundary was determined by a clinical assessment of the previously published literature regarding expected specificity requirements in clinical assessments inclusive of multiple MDDs with access to radiology, pathology, and clinical information, with typical lower-bound confidence intervals for specificities in the high 70s to low 80s [15]. Secondary and exploratory endpoints included specificity comparison between Fibresolve and ECP with multiplicity adjustment and analysis in key subgroups, including (1) cases with a CT slice thickness ≤ 3 mm (as defined by FDA requirements around CT scan quality in final ILD assessment); (2) cases for which the ECP did not positively diagnose IPF (e.g., cases without typical UIP pattern by CT); and (3) sub-analyses by baseline stratification, including CT manufacturer, clinical site, and demographic factors. The key subgroup of interest from a diagnostic perspective was patients with a CT scan slice thickness ≤ 3 mm and clinical and CT scan characteristics not meeting the non-invasive criteria for an IPF diagnosis, for which diagnostic yield is the key metric.

## 3. Results

### 3.1. Baseline Characteristics

**Figure 3:**
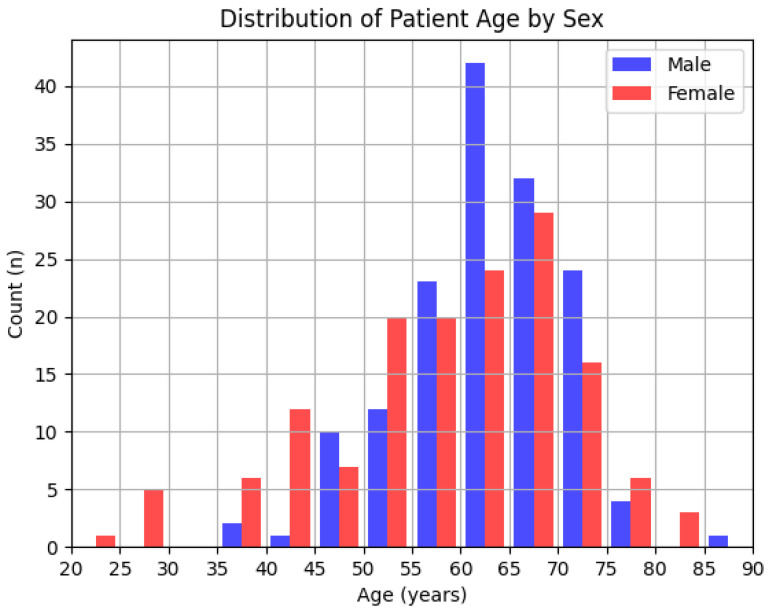
Histogram of age and sex distribution across all cases.

The baseline demographic and clinical characteristics were typical of those previously reported in the literature in ILD and IPF (Table 1) [24,25]. The median age across the full population was 62 years old, 65 years old for IPF patients, and 60 years old for non-IPF patients (*p* = 0.0003). Sex was nearly evenly split with grossly similar age distribution (Figure 3). Surgical tissue results were available for 94.7% of patients. Follow-up times ranged from 0 to 2058 days, with a median of 213 days. The median percent predicted forced vital capacity (ppFVC) was 67.0% across the full population, 63.0% for IPF patients, and 70.5% for non-IPF patients (*p* = 0.0097). Follow-up for living/death status was available for 48.7% of patients. The 50-case subset was similarly represented in all available measures.

**Figure 4:**
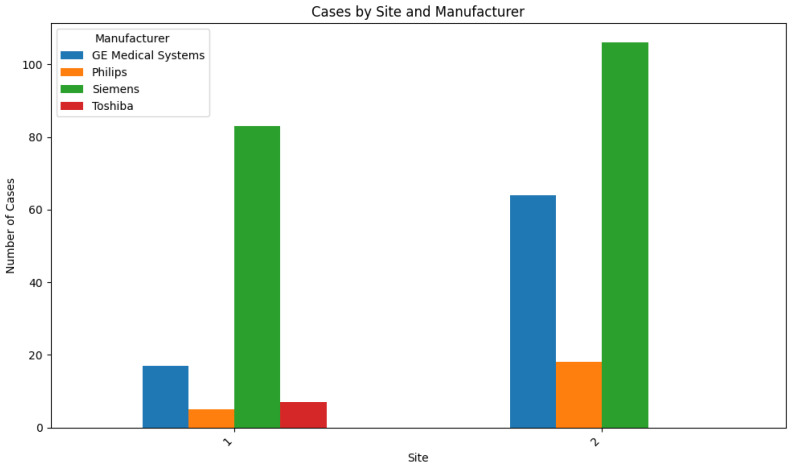
Histogram of site and manufacturer distribution across all cases.

Regarding site and technical characteristics, case distribution for the two sites was 62.7% and 37.3%, respectively (Table 2). The two most common CT manufacturers in practice predominated (GE, Boston, MA, USA; Siemens, Munich, Germany), with two others (Philips, Amsterdam, The Netherlands; Toshiba, Tokyo, Japan) included in smaller numbers (Figure 4). A total of 23 different individual scanner models were included across the clinical sites. Acquisition protocols varied by site, including the use of skip imaging and inclusion of scan series acquired in a prone position, and as a result, contiguous CT slice thickness ranged from 1 to 5 mm. Precise CT reconstruction kernel varied by scanner at each site, with the “sharpest” available selected for automatic processing by the software.

### 3.2. Final Ground Truth Diagnosis

Positives were defined as cases with a final Ground Truth/Reference Standard Diagnosis of IPF. Negatives included the following categories: unclassifiable ILD (UILD), chronic hypersensitivity pneumonitis (CHP), nonspecific interstitial pneumonia (NSIP), cryptogenic organizing pneumonia (COP), connective-tissue-disease-associated ILD (CTD-ILD), desquamative interstitial pneumonia (DIP), eosinophilic granulomatosis with polyangiitis (EGPA), sarcoidosis, berylliosis, respiratory bronchiolitis–interstitial lung disease (RB–ILD), chronic eosinophilic pneumonia (CEP), lymphocytic interstitial pneumonia (LIP), and No ILD (i.e., cases suspicious for ILD but without a final ILD diagnosis; typically, severe emphysema, extensive lung cancer, etc.). Per the reference standard, IPF was the final diagnosis in 27.7% of cases (Table 3). A diagnosis of unclassifiable ILD (UILD) was the second most common at 18.0%, followed by chronic hypersensitivity pneumonitis at 16.7% and nonspecific interstitial pneumonia at 10.7%. The median follow-up time from initial assessment to final diagnosis after surgical biopsy was 213 days.

### 3.3. Performance Assessments

The binary outputs (i.e., positive or negative for a diagnosis of IPF) of Fibresolve assessing the CT scans only and the ECP and smaller CPs assessing the full clinical case information including the CT scan at time zero were obtained and compared to the final subsequent Ground Truth Diagnosis. The Fibresolve system met pre-specified co-primary endpoints of sensitivity superior to ECP and of a specificity statistically and significantly greater (*p* = 0.0007) than the non-inferior boundary of 80.0% (Table 4). In the full 300 cases, assessing at the initial time point and including both dedicated and non-dedicated lung CT protocols, Fibresolve sensitivity was 41.0% versus an ECP sensitivity of 13.3%. The Fibresolve classifier’s specificity was not statistically or significantly different compared to the ECP and within the pre-specified target performance range, though it did trend lower than the ECP. In the key subgroup of interest, CT slice thicknesses ≤ 3 mm and clinical and CT scan characteristics that did not meet the non-invasive criteria for an IPF diagnosis (e.g., indeterminate cases)—Fibresolve operating as a “digital biopsy”-type tool—achieved a diagnostic yield of 53.1% for IPF at 85.9% specificity. There were no statistically significant differences in the performance of Fibresolve by patient age, race or ethnicity, smoking history, clinical site, CT manufacturer, or follow-up period.

Fibresolve sensitivity was superior to all four clinical panels within the 50-case subset. Sensitivity for the panels ranged from 0% to 28.6%, and specificity ranged from 91.7% to 100% in the subset, while Fibresolve sensitivity was 57.1% and its specificity was 88.9% (Table 5).

Within the 50-case subset, agreement was highest (substantial; κ = 0.658) between the ECP and the first of the smaller clinical panels (i.e., second most experienced). Otherwise, agreement between the panels ranged from “no agreement” (κ < 0) to “fair agreement” (κ of 0.21–0.40).

Given that most of the prior literature assessing diagnostic utility in ILD and IPF excludes patients with a final diagnosis of UILD, the assessment was also made in the subgroup excluding that population in this study. Sensitivity was unchanged, and specificity increased for both Fibresolve and ECP in the cases exclusive of those with UILD diagnosis. Results showed the statistically significant superiority of Fibresolve versus ECP for sensitivity and no statistical difference for specificity versus ECP in non-UILD cases.

### 3.4. Analysis of Error Cases

Post hoc assessments were made to analyze the “error” cases for Fibresolve, including false positives and false negatives. Among false positives, UILD and NSIP were the most common final diagnoses, making up 72.4% of false positives for Fibresolve. Similarly, UILD made up 75.0% of false positives for the ECP. Regarding false negatives, there was no statistically significant difference in age, averaging 64 years old for both true positives and false negatives. A statistically significant difference in baseline ppFVC was noted with a median ppFVC of 54.0% for true positives versus 64.5% for false negatives (*p* = 0.02), though the range of ppFVC for true positives was 35–97%, and for false negatives it was 24–99%, indicating heavy overlap between the groups.

## 4. Discussion

In this study, we demonstrated the diagnostic performance of the Fibresolve system as an adjunct in predicting the diagnosis of IPF in the pre-invasive setting. This form of adjunctive artificial intelligence (AI) technology has, in recent years, been broadly referred to as a “digital biopsy” or “virtual biopsy” tool, assessing for imaging features not otherwise assessable by the naked eye, but few such tools have been validated in clinical studies with new cases, and none yet with an eye towards an IPF diagnosis itself. This study was completed per protocol. There were no amendments. There were no safety issues in this retrospective-type design. This study met the pre-specified co-primary endpoints of specificity of Fibresolve greater than the non-inferior boundary of 80%, with a final specificity of 87% and a sensitivity of Fibresolve superior to ECP, at 41% versus 13%. No other FDA-authorized technologies exist to make a direct comparison to, which is why a direct clinical comparator was the best available comparator.

In the key subgroup of interest, cases not meeting pre-invasive criteria for IPF diagnosis and with ≤3 mm slice thickness CT (e.g., dedicated lung CT protocols), Fibresolve achieved a diagnostic yield of 53.1% at a specificity of 85.9%. These cases are otherwise considered for surgical biopsy or other invasive testing. Thus, directly translating the performance in this subgroup would correlate with a 53.1% positive diagnosis rate in this challenging patient population, potentially avoiding biopsy while confirming IPF diagnosis in these cases, a result that favorably compares to previously published reports generally estimating the sensitivity of minimally invasive procedures like bronchoalveolar lavage and transbronchial biopsy sensitivity in the 30% range. In addition, follow-up time from the initial assessment to the final diagnosis was a median of 213 days, indicating that the use of Fibresolve at the initial assessment has the potential to substantially reduce the time to diagnosis for a substantial number of patients.

This study met secondary endpoints of specificity greater than the non-inferior boundary of 80% in cases with pathology, overall superiority in sensitivity versus ECP, and no statistical difference in specificity versus ECP with pre-specified multiplicity adjustment, though Fibresolve specificity trended lower. In secondary analysis of the 50-case subset, specifically, Fibresolve was found to be statistically superior in sensitivity to all four panels; 57% for Fibresolve versus the 0–29% range for the panels. These findings suggest additional value in the context of use for pulmonologists and radiologists who care for ILD patients in relatively less specialized settings.

This study demonstrated a 3.2-fold improvement in the sensitivity of Fibresolve versus ECP overall, despite an unfavorable head-to-head study design for the software. The tested version of the software (version 1.0) analyzed only CT images, while the highly trained ECP reviewed imaging, clinical, demographic, and laboratory data in a group setting. The ECP was able to maintain a high specificity in adhering to the ATS Guidelines. Fibresolve specificity was somewhat lower but remained above the target threshold and in line with the clinically acceptable performance of other tools in the field, especially given known inter-MDD variance [12]. An analysis of false positives from Fibresolve revealed that 45% were ultimately given an UILD diagnosis. Such a diagnosis indicates that no consensus could be reached by the reference standard-assigning clinicians, even with access to follow-ups and surgical pathology. The second largest group of false positives were cases of NSIP. The lines between IPF and fibrotic NSIP have become blurred in recent years, and such differentiation is anticipated to become less critical with time, as treatment strategies converge between IPF and fibrotic NSIP [26].

There are no clear comparators for this particular type of technology in ILD (i.e., software-only), and no other FDA-authorized diagnostic technology was available in this field at the time of this composition. No other published software tools in this area have specifically targeted the differentiation of a diagnosis of IPF from other ILDs; a variety of tools have been described that mimic a radiologist assessment of UIP patterns but without surgical and clinical diagnosis to provide Ground Truth for assessment of accuracy. The main relevant comparators in the literature are clinicians, as assessed in this study, as well as minimally invasive procedures, including transbronchial biopsy and bronchoalveolar lavage, the former of which is reported at 33.4–38.9% diagnostic yield [5]. While head-to-head comparisons across studies must be reviewed with caution, Fibresolve’s 53.1% diagnostic yield compares favorably to these minimally invasive procedures, with the added benefit of zero additional procedural risk. Outside of ILD/IPF but within the field of machine learning, one other conceptual comparator for the study design, pre-invasive use case, and results is FFR-CT (Heartflow; Mountain View, CA, USA). This standalone CT imaging analysis algorithm is used to determine the likelihood of clinically significant coronary artery disease (CAD) in the pre-invasive catheterization setting for patients with clinical signs of CAD. In a pivotal study validating that technology, targeting maintenance of sensitivity while increasing specificity (a parallel to Fibresolve, improving sensitivity while maintaining specificity), the sensitivity was 86.3% (CI: 77.0–92.1%) and the specificity was 78.7% (CI: 72.1–84.2%) versus a clinical assessment comparator sensitivity of 93.8% (CI: 86.2–97.3%) and specificity of 33.9% (CI: 27.3–41.2%) [27]. Though Fibresolve and FFR-CT assess different diseases with differing computer science methods, both tools utilize standalone CT imaging analysis algorithms to help drive improved assessments of patients in a pre-invasive setting. The PUFAIR study results demonstrated Fibresolve’s capacity to maintain a similar specificity while boosting sensitivity in this setting.

Multiple subgroups in the PUFAIR study were analyzed. Two additional key subgroups analyzed included cases excluding the diagnosis of UILD (unclassifiable ILD; i.e., even with follow-up and pathology, the reference standard process was unable to classify the disease) and cases excluding those selected as “IPF Confirmed” by the ECP (i.e., leaving the most challenging cases), maintaining high specificity and increased sensitivity in these groups. These results are of particular importance, as one of the areas of greatest potential utility for Fibresolve is in cases not otherwise non-invasively confirmable by clinicians.

Other subgroups analyzed included clinical and demographic splits by age range, race, ethnicity, and smoking history; geographic/care characteristic splits by clinical site, follow-up period, and time-to-death; and technical imaging characteristic splits by CT manufacturer. Notably, performance appeared to vary little by CT manufacturer and clinical site. Additionally, relatively little variation in Fibresolve performance was noted across age ranges, smoking history, and other basic demographic characteristics. These results are of critical importance, as, historically, machine learning software has suffered from significant challenges related to lack of generalizability, or the ability for the software to perform well across new sites and technical characteristics of underlying data; consistency in Fibresolve performance indicates validated generalizability and, therefore, greater potential for broad utility.

The limitations of this study included a limited number of clinical sites (2), number of ECPs (1), number of CPs (3), data age range, and application of the 2018 ATS Guidelines. Fibresolve was validated with multiple additional clinical sites during development, with consistent discriminatory ability across datasets, confirming generalizability. The ECP was composed of highly qualified ILD specialists with pulmonology and radiology backgrounds who were instructed to follow guidelines in the diagnosis of IPF, but even MDDs are known to have significant levels of inter-MDD discordance. Assessments for variability were made with three additional community panels in the case subset, giving a consistent range of performance across these groups. Agreement between the panels was generally “poor” to “fair”, consistent with the published literature, again highlighting the challenges associated with inter-clinician variability. Data were collected from sources dating from 2005 to 2018, implicating potential data heterogeneity given evolving technologies (e.g., new CT scanners, and changes in practice standards). In the study itself, the 2018 ATS Guidelines were used by the ECP and CPs, consistent with practice standards in 2021 during the study execution, but the 2022 ATS Guideline updates could impact the study results somewhat.

The precise reasons for false negatives are unknown: cases may be too early in the disease course, cases may overlap to a substantial degree with other diagnoses, or contributors to diagnosis may not be primarily driven by CT information. Notably, while ppFVC was slightly lower for Fibresolve true positives, the range was wide and overlapped with the distribution of false negatives, suggesting disease severity is only one small factor in the contribution to diagnostic determination. Finally, the distribution of ILD subtypes can vary significantly. In general, the breadth of represented non-IPF diagnoses was within ranges provided in the literature; however, the percentage of cases with a final diagnosis of UILD was at the upper-end of typical at 18% (some clinical sites fall as low as 5%), and the percentage of cases with a final diagnosis of IPF was at the lower end of typical at 28% (can vary from 30–60%, with ~35–45% being typical). A lower prevalence of UILD would likely improve the specificity for both Fibresolve and the panels. A higher rate of IPF would improve the PPV for Fibresolve and the panels. As such, it may be expected to see a range of absolute performance depending on the precise distribution of IPF cases, but with Fibresolve performance metrics relative to clinical experts preserved.

Implications and Actions Needed

Overall, this study was completed per protocol, met its co-primary endpoints, and demonstrated data in support of the potential use of Fibresolve to predict the diagnosis of IPF in a pre-invasive setting for patients undergoing work up for ILD. It is expected that the two primary values of the tool may include (1) increased sensitivity for IPF in non-expert settings, helping enhance referral patterns to MDDs for comprehensive assessment, and (2) improved diagnostic yield for IPF in cases without typical UIP patterns, with the potential to help reach diagnosis via non-invasive workflows. The next steps for the system are focused on building clinical experience in day-to-day clinical workflow across diverse settings and continuing the refinement of machine learning algorithm performance with time.

## 5. Conclusions

These results demonstrate that in combination with standard clinical assessment, Fibresolve can serve as an adjunct in the diagnosis of IPF in a pre-invasive setting. Specifically, this tool is demonstrated to have the greatest potential use in two key circumstances: (1) as a tool for enhancing referral patterns to MDDs for comprehensive assessment during the initial ILD/IPF identification phase, and (2) as a “digital biopsy” tool for improving diagnostic yield for non-invasive diagnosis of IPF, as differentiated from other ILDs. Beyond ILD/IPF, the underlying technology follows a highly generalizable structure, suggesting that the same approach may be useful in a wide array of disease areas to further improve non-invasive diagnosis in challenging diseases.

## 6. Patents

Provisional patents are pending for the underlying software technology.

## Figures and Tables

**Table 1:** Complete dataset demographic and clinical characteristics.

-	Full Dataset—% (*n*)	50-Case Subset—% (*n*)
**Age**	-	-
≤40	5.0 (15)	2.0 (1)
41–50	10.0 (30)	12.0 (6)
51–60	28.0 (84)	28.0 (14)
61–70	41.3 (124)	42.0 (21)
>70	15.7 (47)	6.0 (6)
**Sex**	-	-
Female	49.7 (149)	50.0 (25)
Male	50.3 (151)	50.0 (25)
**Race**	-	-
American Indian or Alaskan Native	1.0 (3)	2.0 (1)
Asian	0.3 (1)	0.0 (0)
Black or African American	10.3 (31)	10.0 (5)
Multi-race	0.7 (2)	0.0 (0)
Unknown	2.0 (6)	4.0 (2)
White	85.7 (257)	84.0 42
**Ethnicity**	-	-
Hispanic or Latino	9.0 (27)	8.0 (4)
Not Hispanic or Latino	88.7 (266)	90.0 (45)
Chooses not to disclose	2.3 (7)	2.0 (1)
**Pathology**	-	-
1 (Surgical Tissue Recorded)	94.7 (284)	96.0 (48)
0 (None Recorded)	5.3 (16)	4.0 (2)
**Smoking History**	-	-
Yes	69.0 (207)	74.0 (37)
No	31.0 (93)	26.0 (13)
**Follow-Up Time**	-	-
0–150 Days	28.3 (85)	36.0 (18)
151–300 Days	40.3 (121)	30.0 (15)
300+ Days	31.3 (94)	34.0 (17)
**Lung Function**	-	-
ppFVC * < 50%	19.7 (59)	16.0 (8)
ppFVC 50–75%	36.0 (108)	40.0 (20)
ppFVC > 75%	34.7 (104)	36.0 (18)
**Mortality**	-	-
Alive at last time point	32.0 (96)	26.0 (13)
Dead at last time point	16.7 (50)	18.0 (9)
Unknown	51.3 (154)	56.0 (28)

* ppFVC = percent predicted forced vital capacity; not available for 15 patients.

**Table 2:** Dataset technical characteristics including clinical site, CT imaging, and follow-up characteristics.

-	Full Dataset—% (*n*)	50-Case Subset—% (*n*)
**Clinical Sites**	-	-
Site #1	37.3 (112)	36.0 (8)
Site #2	62.7 (188)	64.0 (1)
**Manufacturer**	-	-
GE Medical Systems	27.0 (81)	26.0 (13)
Philips	7.7 (23)	12.0 (6)
Siemens	63.0 (189)	60.0 (30)
Toshiba	2.3 (7)	2.0 (1)
**Slice Thickness**	-	-
1 mm	14.7 (44)	16.0 (8)
1.25 mm	3.7 (11)	2.0 (1)
1.5 mm	12.7 (38)	6.0 (3)
2.0 mm	1.3 (4)	6.0 (3)
2.5 mm	1.3 (4)	2.0 (1)
3.0 mm	13.3 (40)	16.0 (8)
3.75 mm	0.7 (2)	0.0 (0)
4.0 mm	0.3 (1)	0.0 (0)
5.0 mm	53.3 (160)	52.0 (26)

**Table 3:** Dataset breakdown by final clinical diagnosis of the MDD.

-	Full Dataset—% (*n*)	50-Case Subset—% (*n*)
**IPF**	27.7 (83)	28.0 (14)
**Not IPF**	72.3 (217)	72.0 (36)
- UILD	18.0 (54)	24.0 (12)
- CHP	16.7 (50)	12.0 (6)
- NSIP	10.7 (32)	8.0 (4)
- COP	7.7 (23)	8.0 (4)
- CTD-ILD	4.3 (13)	6.0 (3)
- DIP	3.0 (9)	4.0 (2)
- EGPA	3.0 (9)	0.0 (0)
- Sarcoid	2.7 (8)	4.0 (2)
- No ILD	1.3 (4)	2.0 (1)
- Berylliosis	1.3 (4)	2.0 (1)
- RB–ILD	1.0 (3)	0.0 (0)
- CEP	0.3 (1)	0.0 (0)
- LIP	0.3 (1)	2.0 (1)

**Table 4:** Overview comparison of key diagnostic performance of Fibresolve and Expert Clinical Panel.

-	Expert Clinical Panel in All Cases	Fibresolve in All Cases	Fibresolve in Key Subgroup *
Cases	300	300	124
Sensitivity	13.3% [CI: 6.8–22.5]	41.0% [CI: 30.3–52.3] **	53.1% [CI: 41.3–64.9]
Specificity	96.3% [CI: 92.9–98.4]	86.6% [CI: 81.4–90.9] ***	85.9% [CI: 76.7–92.6%]

* CT slice thickness ≤ 3 mm and without pre-invasive IPF diagnosis (e.g., indeterminate cases), ** *p* = 0.0007, *** There was no statistically significant difference with pre-specified multiplicity adjustments, though Fibresolve trended toward a somewhat lower specificity above the 80% target threshold.

**Table 5:** Comparison of diagnostic performance of Fibresolve and all panels in the 50-case subset cases.

-	Sensitivity	Specificity
Fibresolve	57.1%	88.9%
Expert Clinical Panel	0%	94.4%
Clinical Panel #1	0%	97.2%
Clinical Panel #2	28.6%	91.7%
Clinical Panel #3	14.3%	100%

## Data Availability

All publicly available data sources are listed in the acknowledgements and available by request from the dataset owners.
